# Individual behavioral profiling as a translational approach to assess treatment efficacy in an animal model of post-traumatic stress disorder

**DOI:** 10.3389/fnins.2022.1071482

**Published:** 2022-12-22

**Authors:** Ishita Sarkar, Maja Snippe-Strauss, Adi Tenenhaus Zamir, Amir Benhos, Gal Richter-Levin

**Affiliations:** ^1^Sagol Department of Neurobiology, University of Haifa, Haifa, Israel; ^2^School of Psychological Sciences, University of Haifa, Haifa, Israel; ^3^The Integrated Brain and Behavior Research Center, University of Haifa, Haifa, Israel

**Keywords:** PTSD, individual behavioral profiling, fluoxetine, treatment-responders, excitation-inhibition balance, animal model of PTSD

## Abstract

A major challenge in treating post-traumatic stress disorder (PTSD) continues to be the large variability in responsiveness to pharmacotherapy. Only 20–30% of patients experience total remission to a specific treatment, while others demonstrate either partial remission or no response. However, this heterogeneity in response to pharmacotherapy has not been adequately addressed in animal models, since these analyze the averaged group effects, ignoring the individual variability to treatment response, which seriously compromises the translation power of such models. Here we examined the possibility of employing an “*individual behavioral profiling*” approach, originally developed to differentiate between “affected” and “exposed-unaffected” individuals in an animal model of PTSD, to also enable dissociating “*responders*” or “*non-responders*” after SSRI (fluoxetine) treatment. Importantly, this approach does not rely on a group averaged response to a single behavioral parameter, but considers a cluster of behavioral parameters, to individually characterize an animal as either “*responder*” or “*non-responder*” to the treatment. The main variable to assess drug efficacy thus being the proportion of “*responders*” following treatment. Alteration in excitatory/inhibitory (E/I) balance has been proposed as being associated with stress-related psychopathology. Toward a functional proof of concept for our behaviorally-based characterization approach, we examined the expression patterns of α1 and α2 subunits of GABA_A_ receptor, and GluN1 and GluN2A subunits of the NMDAR receptor in the ventral hippocampus, as well as electrophysiologically local circuit activity in the dorsal dentate gyrus (DG). We demonstrate that with both parameters, treatment “*responders*” differed from treatment “*non-responders*,” confirming the functional validity of the behavior-based categorization. The results suggest that the ability to respond to fluoxetine treatment may be linked to the ability to modulate excitation-inhibition balance in the hippocampus. We propose that employing the “*individual behavioral profiling*” approach, and the resultant novel variable of the proportion of “recovered” individuals following treatment, offers an effective translational tool to assess pharmacotherapy treatment efficacy in animal models of stress and trauma-related psychopathology.

## Introduction

While treatment efficacy in humans is measured by comparing an individual’s behavioral and physiological traits to an averaged healthy population, the accepted approach of measuring efficacy of pharmacological treatments in animal models of psychiatric disorders refers to averaged group results (Katz and Hersh, 1981; Bodnoff et al., 1989; Rudolph and Feiger, 1999; Dulawa et al., 2004). This contradiction is particularly evident in studies assessing the effects of pharmacotherapy in animal models of post-traumatic stress disorder (PTSD) and the actual response rate to pharmacotherapy in PTSD patients. For instance, Wilson et al. (2014), reported that chronic sertraline treatment in animal model of PTSD did not alleviate anxiety-like behavior as evinced by no increase in exploration of the open arms in elevated-platform test after treatment (Wilson et al., 2014). Similarly, previous work from our lab had shown that chronic fluoxetine treatment failed to rescue PTSD-like symptoms in adult rats post-trauma (Ariel et al., 2017). In both studies, the behavioral response to sertraline or fluoxetine was reported as an averaged group effect of the treated animals. However, humans statistics suggest that while the majority of PTSD patients do not respond to treatment, a considerable fraction of 30–40% patients do respond to pharmacotherapy (Ravindran and Stein, 2010) and a significant minority of 20–30% of patients attain complete remission (Steckler and Risbrough, 2012). This individual variability in treatment response is obscured in animal models using group averages, thus limiting their translational power.

A way to overcome this limitation, however, can be if each animal is profiled individually based on their behavior. The concept of profiling animals individually based on certain behavioral “cut-off” values was first established by Cohen et al. (2007) and later modified by our lab to categories trauma-exposed animals as PTSD-affected or PTSD-unaffected, in reference to the behavior of a control population (Ardi et al., 2016). In the current study, we aimed to adapt the *individual behavioral profiling (IBP)* approach in order to address the heterogeneity in treatment response in an animal model of PTSD (Richter-Levin et al., 2015). We administered chronic fluoxetine treatment to the PTSD-affected animals and employed the IBP approach to classify them as treatment *responders* or non-*responders*.

Fluoxetine belongs to the class of selective serotonin reuptake inhibitors (SSRIs) and is used as a first line (off-label) medication for treating PTSD patients (Management of Post-Traumatic Stress Working Group, 2010). However, effectiveness of fluoxetine is dubious since different studies either acclaim it as beneficial in civilians and veterans (Connor et al., 1999) or report its inefficiency (Martenyi et al., 2007). Probably, part of the confusion regarding the efficacy of SSRIs results from the fact that, since only about 30% of patients show full remission, the averaged efficacy of the drug is not optimal. However, this averaged view obscures the efficacy of the drug for those who respond well to the treatment.

Post-traumatic stress disorder is characterized by impaired GABAergic (Girgenti et al., 2021) and glutamatergic transmission (Krystal et al., 2017), especially in the hippocampus. This disruption in the excitation-inhibition (E/I) balance has been extensively investigated in animal models of PTSD. Traumatic stress in juvenility has been reported to induce anxiety-like behavior and altered α2, α5, and γ2 subunits expression of GABA_A_ receptor in the hippocampus (Lu et al., 2017). Increased α1 expression in the ventral hippocampus has been associated with resilience to PTSD-like symptoms in an animal model of PTSD (Ardi et al., 2019). Similarly, a study in predator-exposed rat model of PTSD reported decreased expression of GluN1 subunit of NMDA receptor in the dorsal hippocampus following trauma, which resulted in increased nitric oxide synthase in neurons, which was implicated in several anxiety-disorders (Ayhan et al., 2016). Increased expression of GluN1 and GluN2A subunits of NMDA receptor in the frontal and pre-frontal cortex post trauma was also observed in an acute foot-shock model of PTSD (Bonini et al., 2016). Further, previous research from our lab showed that exposure to trauma and/or re-exposure to trauma reminder increased feed-forward inhibition in the local inhibitory circuits and impaired long-term potentiation (LTP) in the dorsal dentate gyrus (dDG) in rat-model of PTSD (Ardi et al., 2014). Together the evidence suggests that disruption of the E/I homeostasis is one of the major underlying causes of PTSD pathophysiology (Chambers et al., 1999; Bremner et al., 2000; Reul and Nutt, 2008; Meyerhoff et al., 2014; Michels et al., 2014; Rosso et al., 2014).

Fluoxetine is known to influence E/I balance by altering GABA and glutamate mediated neurotransmission (Fang et al., 2018; Lazarevic et al., 2019; Sheth et al., 2019). It can increase GABA_A_ receptor activity through novel modulatory sites in α1-α6 subunits except α5 (Robinson et al., 2003). Additionally α2 subunit is essential for manifesting anxiolytic effects of fluoxetine (Benham et al., 2017). Fluoxetine also modulates NMDA receptor activity by selectively inhibiting GluN2B containing receptors (Kiss et al., 2012). Chronic fluoxetine administration enhances maturation of synapses and dendritic arborization by the increasing concentration of GluN2A subunits in the PFC (Ampuero et al., 2019).

In the current study we employed a rat model of PTSD that combined juvenile stress and trauma exposure in adulthood (Ardi et al., 2016). This set-up simulates early life adversity that increases the risk of developing psychiatric disorders (Heim and Nemeroff, 2001) and a severe acute trauma that mimics trauma exposure in PTSD patients. In the first set of experiments we employed IBP to classify animals as trauma *affected* or *unaffected*. Trauma *affected* animals were administered chronic fluoxetine treatment in drinking water for a period of 30 days. At the end of the treatment, the animals were categorized as treatment *responders* or *non-responders* according to a second round of IBP (IBP I). As a biochemical proof of concept for IBP I and in order to better understand the influence of fluoxetine on E/I balance, we measured the expression levels of GABA_A_α1, GABA_A_α2 and GluN1, GluN2A receptor subunits in the ventral hippocampus in *responders* and non-*responders*. In the second set of experiments, we checked the flexibility and strength of the IBP approach by incorporating a different set of behavioral parameters. We used IBP approach II to classify animals as trauma *affected* and later distinguished them as treatment *responders* or non-*responders* after fluoxetine treatment. As a testbed for IBP II and to investigate the alteration of E/I balance at neural circuit level, we measured changes in the local circuit activity and LTP in the dDG in treatment *responders* and non-*responders*.

We hypothesized that both sets of IBP would effectively delineate between treatment *responders* and *non-responders*. The percentage of responsivity to fluoxetine would be close to that observed in human PTSD patients receiving SSRIs. We further expected to see differential expression pattern of the GABA_A,_ and NMDAR receptor subunits, and altered electrophysiological properties between *responders* and *non-responders*, which may point to the involvement of altered I/E balance in treatment responsiveness.

## Materials and methods

### Animals

Male Sprague Dawley rats of post-natal day 22 were ordered from Harlan laboratories, Jerusalem. Animals were group housed (22 ± 2°C, 12 h light dark cycle) with food and water ad libitum. Five days of acclimatization period was provided to all animals before the start of the experiments. All experiments were performed according to the NIH guide for care and use of laboratory animals and approved by the University of Haifa ethical committee.

### An animal model of post-traumatic stress disorder

In the current study we employed the previously established rat model of PTSD, which combines exposure to juvenile stress (JVS) with a later exposure to underwater trauma (UWT) in adulthood (Richter-Levin et al., 2015). Briefly, animals were exposed to the juvenile stress protocol (Horovitz et al., 2012) for a period of three consecutive days during post-natal days (PND) 27-29. The protocol is comprised of three different stressors (i) Forced swim (15 min) on day 1, (ii) elevated platform (30 min, 3 times, 1-h break between each session) on day 2, and (iii) restrain stress (2 h) on day 3. After that, the animals were left undisturbed for a period of 30 days. The control group was not exposed to JVS.

On PND 60 the animals were brought and habituated for 5 min in the UWT room. The water filled plastic tank for UWT (diameter 70 cm, height 50 cm, water depth 40 cm, water temperature 22 ± 2°C) was part of the water associated zero maze (WAZM) set-up (Ritov and Richter-Levin, 2014). The animals were allowed 5 min of exploration the WAZM platform. Immediately after that, they were restrained under water, inside the water tank, for 45 s by a special net (20 × 20 × 15 cm). The control groups were not immersed in water and were returned to their cages after exploration.

### Behavioral tests

#### Water associated zero maze

This behavioral test set-up was previously established in our lab to measure anxiety caused by traumatic re-experiencing animals (Ritov and Richter-Levin, 2014). It consisted of an annular platform (90 cm diameter; 10 cm width), made out of black plywood, and joined to the UWT plastic tank. The annular platform had two opposite closed quadrants (with walls 35 cm height) and two open quadrants (with borders 5 mm height). For the tests, rats were first habituated to the room for 5 min and then were placed into one of the open quadrants facing a closed part of the apparatus. Rats were allowed to explore the arena for 5 min. During this time, behavior of the rat was tracked, recorded and analyzed by the Etho-Vision system (Noldus Information Technology, Wageningen, Netherlands).

### Open field test

Open field test was used to measure anxiety-like behavior in animals by analyzing the total distance traveled or time spent in the center of the OFT box (Gould et al., 2009). Animals were first habituated for 5 min to the OFT room before starting the experiment. The rats were then placed at the corner of the open field box (90 cm × 90 cm × 50 cm, dim white light illumination) and allowed to explore the arena freely for 5 min (Avital and Richter-Levin, 2005).

### Social recognition test

Social recognition test measures the social recognition memory of an animal by determining the preference index of the subject animal for an unfamiliar animal over a familiar animal (Eagle et al., 2013; Gur et al., 2014; Jacobs et al., 2016). This test is based on the observations that rodents tend to prefer exploring socially novel partner over familiar (Thor and Holloway, 1982). In this study we measured short-term social recognition memory. SRT was conducted a day following the OFT, in the same arena. At the end of the OFT recording, the animals were habituated with corrals (9 cm in diameter, slotted with holes and covered with Plexiglas) kept in a diagonally opposite direction for 5 min. These corrals were used to place the stimuli for SRT the next day. On the day of SRT, the subject rats were first familiarized to a juvenile conspecific kept in one of the corrals for a period of 15 min. After the familiarization session all the animals were returned to home-cage for 30 min. After the 30 min the subject animal was brought back from its home cage and placed again in the arena for 5 min with the previous familiar juvenile conspecific and a novel unfamiliar juvenile conspecific (Gur et al., 2014). The time spent investigating each corral was recorded and measured using the EthoVision XT8 tracking system (Gur et al., 2014). The position of familiar and unfamiliar animals was changed between animals to minimize positional bias. Preference index for unfamiliar animal in SRT was calculated by using the following formula (Eagle et al., 2013):


(Timespent(unfamiliar)-Timespent(familiar)Timespent(unfamiliar)+Timespent(familiar))x 100

### Elevated plus maze

The EPM set-up consisted of two opposing open (anxiogenic) and closed (anxiolytic) arms which is used to measure anxiety-like behavior in rodents (Walf and Frye, 2007). Animals were brought from their home-cage and habituated to the EPM room for 5 min. The test was carried out by placing the rats in the center of the EPM maze (110 cm × 110 cm, 70 m above the floor; full light illumination) facing an open arm and allowing them to explore the maze freely for 5 min. The behavior of the animal was analyzed by EthoVision XT8 video tracking system (Noldus, Wageningen, Netherlands).

### Fluoxetine administration

Twenty-four-hour water consumption was measured in animals for three consecutive days prior to drug delivery. Fresh solutions were given twice a week using Fluoxetine stock solution of (3 mg/ml) (Vetmarket, Petah tikva, Israel). Animals were weighed before and given fluoxetine at a dose of 10 mg/kg/day in drinking water after calculating the concentration based on their body weight and average daily intake of water (Ariel et al., 2017). The treatment was given for 30 days. Fluoxetine was given in opaque bottles due to its light sensitivity.

### Experiment 1

#### Experimental paradigm I

**FIGURE 1:**
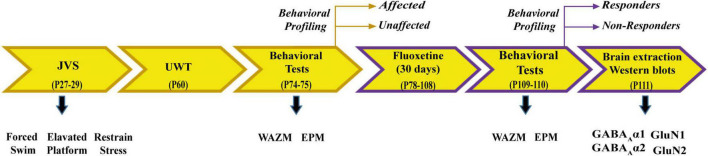
Schematic representation of experimental paradigm 1.

Animals were exposed to JVS at PND 27 and UWT at PND 60. Behavioral tests, including WAZM and EPM, were conducted 2 weeks post UWT to differentiate animals into trauma *affected* or *unaffected*. Trauma *affected* animals were given fluoxetine treatment for a period of 30 days in drinking water. At the end of the treatment, WAZM and EPM test were repeated to classify animals as treatment *responders* or *non-responders*. Animals were sacrificed and brain tissues were harvested at the conclusion of the behavioral tests. Western blot analysis was conducted to check expression of α1, α2 subunit of GABA_A_ receptor and GluN1 and GluN2A subunit of NMDAR in the (i) ventral dentate gyrus (vDG), (ii) ventral CA1 (vCA1), (iii) ventral CA3 (vCA3) (Figure 1).

#### Individual behavioral profiling I

Based on the performance on WAZM and EPM, 7 behavioral parameters that were reflective of explorative activity and anxiety levels were chosen (Supplementary Figure 1). One standard deviation was used to decide the cut-off value for each behavioral parameter based on the performance of the control animals. Animal whose behavioral parameter value was either lower or higher than the cut-off value was considered affected in that specific parameter. Post-UWT, animals which were found to be affected in at least 4 out of 7 parameters were classified as trauma *affected*. Similarly post treatment animals were required to exhibit less than 4 affected parameters to be considered treatment *responders*. The following parameters were used to classify the animals as trauma *affected* and *unaffected* or fluoxetine *responders* and *non-responders*:

i) Total distance traveled in WAZM
ii) Time spent in closed arms of WAZM
iii) Distance traveled in closed arms of WAZM
iv) Distance traveled in open arms of WAZM
v) Total freezing in WAZM
vi) Total freezing in EPM
vii) Anxiety index in EPM closed arm time

(timeinclosedarms+timeincenterTotaltime)x 100

#### Experimental groups

Rats were randomly assigned to one these two groups: *controls* (*n* = 16) and JVS + UWT i.e., *Trauma exposed* (*n* = 93). *IBP 1* was used to classify animals as *trauma-affected* or *trauma-unaffected*. The *trauma-affected*) rats (*n* = 53) were treated with fluoxetine for 30 days. Post-treatment animals were further classified as *treatment responders* or *non-responders*, again based on *IBP 1.* Brain tissue was harvested and the ventral hippocampus dissected out from a subset of these animals. Western-blot experiment was conducted for *responders* which showed complete symptom remission (*n* = 6), *non-responders* (*n* = 4) that continued to be severely affected on all behavioral parameters and unaffected *control* animals (*n* = 5).

#### Brain harvesting and tissue collection

Animals were sacrificed and the brains were snap frozen in dry ice to keep the protein stability intact. On the cryostat, frozen tissue punches were collected (diameter 1 mm, depth 1.5 mm) from vDG, vCA1, and vCA3—7.6 from Bregma, horizontal orientation of the brain.

#### Protein preparation and western blot analysis

One milliliter of lysis buffer was prepared for tissue homogenization (HEPES 10 mM, EDTA 2 mM, EGTA 2 mM, DTT 0.5 mM, protease inhibitor 1:100 dilution, phosphatase inhibitor 1:100 dilution, SDS 0.5% and double distilled water). Hundred microliter of this solution was used to homogenize tissue samples. Protein concentration was estimated using Bradford assay.

Ten microgram of protein was loaded on a 10% SDS-polyacrylamide gel for electrophoresis (SDS-PAGE). The proteins from the gel were transferred to nitrocellulose membrane using semi-dry transfer technique. The proteins on the membrane were visualized using ponceau staining. The membrane was cut according to the molecular weight marker for the protein of interest. The nitrocellulose membrane was washed with double distilled water and 1× TBST to remove ponceau staining. The membrane was later blocked with 5% milk in 1× TBST for 2.5 h. After blocking, the membrane was washed three time with 1× TBST. The membranes were incubated overnight at 4°C with rabbit polyclonal to GABA_A_ α1 1: 5,000 (Abcam—ab33299), rabbit polyclonal to GABA_A_ α2 1: 1,000 (Abcam—ab72445), rabbit polyclonal to GluN1 1:1,000 (Abcam—ab521717), rabbit polyclonal GluN2A 1:1,000 (Abcam—ab169873) or rabbit polyclonal GAPDH 1:10,000 (Abcam—ab9485). The next day, membranes were washed thrice with 1× TBST and incubated for 1 h with Goat α-rabbit secondary antibody 1: 10,000 (Abcam—ab6721). The membranes were washed thrice and developed using ECL plus substrate for chemiluminiscence. Since the molecular weight of α1 and α2 of GABA_A_ receptor are the same, two separate gels were run for each sample. The optical density of the signals was measured using Quantity1 analysis software. The optical density for each band was first normalized to background intensity and then with its respective GAPDH signal. The optical density was then normalized to mean density of the control group for each target protein and region.

### Experiment 2

#### Experimental paradigm 2

**FIGURE 2:**
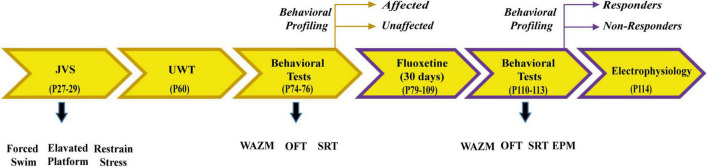
Schematic representation of experimental paradigm 2.

Animals were subjected to the same protocol of JVS at PND 27 and adulthood UWT at PND 60. Two weeks post UWT, animals were categorized into trauma *affected* or *unaffected* based on their behavior in WAZM, OFT, and SRT. Trauma *affected* animals were administered fluoxetine treatment for 30 days in drinking water. At the end of treatment animals were subjected to WAZM, OFT, SRT, and EPM. The animals were behaviorally profiled based on their behavior in WAZM, EPM, and SRT. At the end of the behavioral battery the animals were taken for in-vivo electrophysiological study to investigate local circuit activity and long term potentiation in the dorsal dentate gyrus (dDG) (Figure 2).

#### Individual behavioral profiling 2

In order to classify animals as trauma *affected* or *unaffected* post UWT, behavioral parameters were selected from WAZM, SRT, OFT. The behavioral parameters of WAZM, SRT, EPM were used to classify animals as treatment *responders* or *non-responders*. The rationale for replacing OFT with EPM at the post treatment time point was the observation that control animals did not remain curious enough to explore the same arena on their second exposure to OFT. We replaced the two parameters of OFT that measured explorative activity and anxiety-like behavior, i.e., (i) *total distance traveled* and (ii) *time spent in OFT center* at the post trauma time point with two similar readouts from EPM at the post-treatment time point namely (i) *total distance traveled in EPM* and (ii) *Anxiety index EPM open arm time*. Similar to IBP1, behavioral cut-off values based on one standard deviation from the performance of the control animals were determined for each parameter and for each time point. Animals affected in at least 4 out of 7 parameters were considered trauma *affected* post trauma and those who showed recovery in at least 4 parameters after fluoxetine treatment were considered treatment *responders*.

The following parameters were used post-trauma to classify the animals as trauma *affected* and *unaffected*.

i) Distance traveled in closed arms of WAZM
ii) Time spent in open arms of WAZM
iii) Distance traveled in open arms of WAZM
iv) Total freezing in WAZM
v) Preference index of SRT
vi) Total distance traveled in OFT
vii) Time spent in OFT center

In addition to the first five parameters mentioned above the following two parameters of EPM were used at the post-treatment time point.

i) Total distance traveled in EPM
ii) Anxiety index in EPM closed arm time

#### Experimental groups

This experimental set of animals was divided into two groups: *controls* (*n* = 16) and *Trauma exposed* (*n* = 72). *IBP 2* was used to classify animals as *trauma-affected* or *trauma-unaffected*. Fluoxetine was administered to *trauma-affected* rats (*n* = 35) for a duration of 30 days. IBP 2 was used to classify animals as *treatment responders* or *non-responders* post-fluoxetine treatment. These animals were later subjected to electrophysiological tests to measure local circuit activity and long-term potentiation in dDG. For measuring local circuit activity, the following number of animals were analyzed: *controls* (*n* = 14), *responders* (*n* = 12), and *non-responders* (*n* = 18). Animals which either died during the protocol, were outliers, or did not have stable baseline activity were excluded from the analysis. For the LTP measurement the data of *controls* (*n* = 11), *responders* (*n* = 8), and *non-responders* (*n* = 13). Animals were excluded from analysis if baseline was found unstable.

#### Electrophysiology

Animals were anesthetized (40% urethane, 5% chloral, hydrate in saline, max. 0.5 ml/100 g i.p.) and placed in a stereotaxic apparatus. Body temperature (maintained at 37 ± 0.5°C with Homeothermic Blanket System, Harvard apparatus, Dover, MA, USA) and level of anesthesia were monitored and adjusted if necessary during the whole experiment. The scalp was opened and of small bur holes (1 mm diameter) were drilled. A glass recording electrode (tip diameter 2–5 μm, filled with a 2 M NaCl solution) was inserted into the dorsal DG (coordinates: –4 mm AP, 2.5 mm ML from Bregma and appr. –3.7 mm DV from brain surface) and a bipolar 125 μm stimulating electrode was placed in the perforant path (PP; coordinates: 8.0 mm AP and 4 mm ML from bregma, –3 mm DV from brain surface. The final depth of the electrodes was adjusted to yield maximal excitatory postsynaptic potentials (EPSP), evoked by a single pulse delivered to the ipsilateral PP (0.1 ms duration; rectangular monophasic). The characteristic features of the depth profile of the DG response thereby served as an indicator for correct electrode location. Evoked field potentials were amplified (×1,000), bandpass filtered at 0.1–1,000 Hz (AM-Systems amplifier), digitized at 10 kHz (CED, Cambridge, UK) and stored to disk for off-line analysis using Spike-2 software (version 4.24, CED, Cambridge, UK), where the field EPSP slope and the population spike (PS) amplitude were measured.

After electrode insertion, recordings were allowed to stabilize for 20 min before recording of an *input-output curve* commenced (stimulation intensities from 0.4 to 3.2 mA, 0.2 ms duration) to determine the baseline stimulation intensity (40% of maximal field potential response evoked). After 30 min of *baseline* recording (at 0.1 Hz), different protocols were applied to determine local circuit activity:

*a) Frequency-dependent inhibition (FDI)*: 10 pulses at 0.1 Hz followed by 10 pulses at 1 Hz. The average of EPSP and PS slope of 10 pulses at 0.1 Hz was compared to the average of 10 pulses given at 1Hz (Maroun and Richter-Levin, 2002).
*b) Paired-pulse inhibition (PPI)*: The PPI protocol was used to measure feed-back inhibition or facilitations by giving 5 sets of two pulses with an inter-stimulus interval (ISI) of 15, 30, and 80 ms. Five minutes of baseline stimulation was given before each protocol. The responses to the 2nd pulse were averaged and compared to the average responses to the baseline pulses.

For each protocol, the inhibition strength was expressed as percentage of the baseline response, for the population spike amplitude and the EPSP slope, respectively.

For measuring LTP, baseline recordings of EPSP were assessed during 20 min, at frequency of 0.1 Hz. LTP was induced by theta burst stimulation (TBS) (Ardi et al., 2014): three sets of 10 trains, each with 10 pulses (100 Hz), were administrated with 200 ms inter-train interval, and 1 min inter-set interval. LTP was recorded for 60 min after TBS.

### Statistical analysis

Data is presented as mean ± SEM. The normality of each data set was analyzed using Shapiro–Wilk test. If the data was found to be normally distributed, One-way ANOVA was employed. Bonferroni’s *post-hoc* comparison was used if the *p* value was found to be significant for ANOVA. In case the data was found to be distributed not normally, Kruskal–Wallis test was employed. Dunn’s *post-hoc* comparison was used if the p value was found to be significant. Distribution of trauma *affected* and *unaffected* animals post UWT was compared with that of *controls* using Fisher exact test. Distribution of *affected unaffected* and *responder* and *non-responders* between experiment 1 and 2 were compared using Fisher exact test.

## Results

### Result experiment 1

#### Individual behavioral profiling-1 effectively differentiated trauma-affected animals from trauma-unaffected animals

**FIGURE 3:**
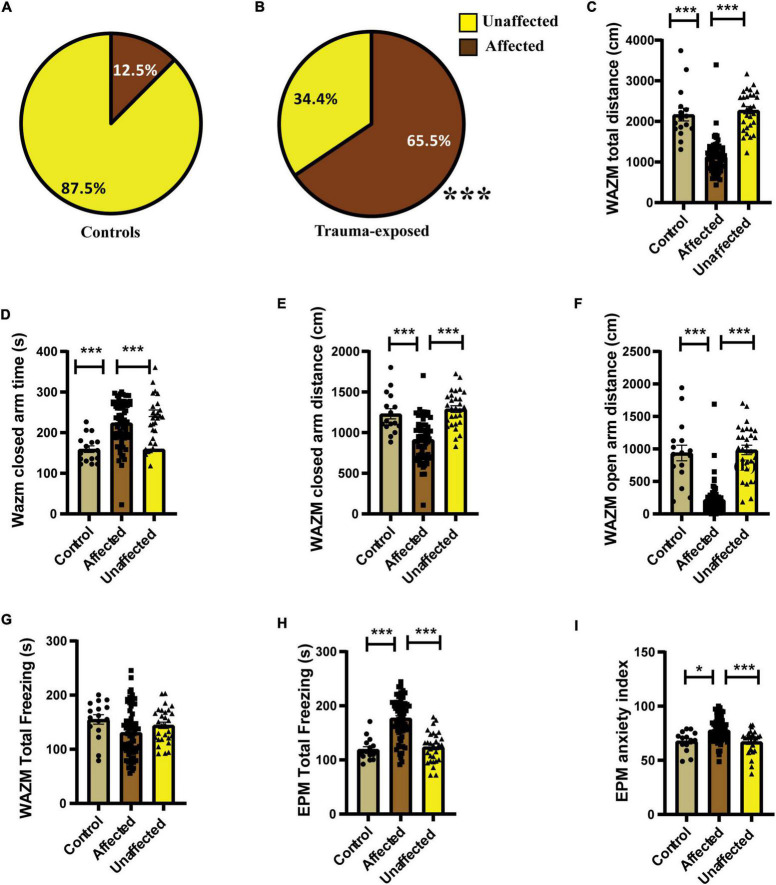
IBP1 classified animals into *affected* or *unaffected* in different groups. **(A)** Percentage distribution of *affected* animals and *unaffected* animals in *control* group. **(B)** Percentage of *affected animals* was found to be significantly higher in *trauma-exposed* animals than *controls* at 2 weeks post UWT [(Fisher exact test, p*** < 0.001); *Control* (*n* = 16), *trauma-exposed* (*n* = 93)]. **(C)** Total distance traveled by trauma *affected* animal was less than *controls* and *unaffected* animals [Kruskal–Wallis test (H (3) = 67.36, *p* < 0.0001); Dunn’s *post-hoc* comparison, p*** < 0.001]. **(D)** Time spent in the closed arms of WAZM was more by the trauma *affected* animals than *controls* and trauma un*affected* animals [Kruskal–wallis test (H (3) = 34.53, *p* < 0.0001); Dunn’s *post-hoc* comparison, p*** < 0.001]. **(E)** Distance traveled in the closed arms of WAZM was least in the trauma *affected* animals [Ordinary-One-Way ANOVA [*F*_(2,106)_ = 27.31, *p* < 0.0001]; Bonferroni *post-hoc* comparison p*** < 0.0001]. **(F)** Distance traveled in the open arms of WAZM was least in the trauma *affected* animals [Kruskal–Wallis test (H (3) = 61.24, *p* < 0.0001); Dunn’s *post-hoc* multiple comparison p*** < 0.0001]. **(G)** Total freezing in WAZM was not different between *controls*, trauma *affected* and *unaffected* animals [Ordinary one-way ANOVA (*F*_(2,106)_ = 2.686, *p* = ns)]. **(H)** Total time spent freezing in the EPM was higher in the trauma *affected* than *controls* and trauma *unaffected* [Ordinary one-way ANOVA (*F*_(2,106)_ = 35.13, *p* < 0.0001); Bonferroni’s multiple comparison p*** < 0.0001]. **(I)** Anxiety index in EPM was highest in the trauma *affected* [Kruskal–Wallis test (H (3) = 19.06, *p* < 0.0001); Dunn’s multiple comparison p* < 0.05, p***0.001]. In all the behavioral parameters of WAZM and EPM no significant difference was observed between the *controls* and trauma *unaffected* individuals. All values are represented as mean ± SEM. *Controls* (*n* = 16), trauma *affected* (*n* = 64), trauma *unaffected* (*n* = 29).

Individual behavioral profiling-1 revealed that not every animal that was exposed to JVS+UWT was trauma *affected*. Nevertheless, the majority of *trauma-exposed* animals developed PTSD-like symptoms (∼65.6%), while around (34.6%) of the animals remained *unaffected* (Figure 3B). Proportion of trauma *affected* individuals was found to be significantly higher in the *trauma-exposed* group than the *controls* group (Fisher exact test, *p* < 0.0001). Only 12.5 % animals of the *control* group were found to be *affected* (Figure 3A).

The average group effect on behavior parameters indicated that the *trauma-affected* animals were severely affected in most of the behavioral measures. (i) *Total distance traveled in WAZM* revealed significant main effect of the groups [H (3) = 67.36, *p* < 0.0001] as measured by Kruskal–Wallis test. Dunn’s *post-hoc* comparison further revealed that the *trauma-affected* animals traveled significantly less distance than both the *control* (*p* < 0.0001) and *unaffected* animals (*p* < 0.0001) (Figure 3C). (ii) *Time spent in the closed arms of the WAZM* was compared across the three groups and Kruskal–Wallis test showed significant difference between groups [H (3) = 34.53, *p* < 0.0001]. Dunn’s *post-hoc* multiple comparison verified that the trauma *affected* animals spent more time in the anxiogenic closed arms of WAZM than *control* (*p* < 0.0001) and *unaffected* (*p* < 0.0001) animals (Figure 3D). (iii) *Distance traveled in closed arms of the WAZM* was found to be significantly different across the groups as shown by Ordinary-One-Way ANOVA [*F*_(2,106)_ = 27.31, *p* < 0.0001]. Bonferroni *post-hoc* comparison showed that the *affected* animals traveled less in the WAZM closed arms than both *control* (*p* < 0.0001) and *unaffected* (*p* < 0.0001) animals (Figure 3E). Significant difference between the three groups was found in the iv) *distance traveled in open arms* by the Kruskal–Wallis test [H (3) = 61.24, *p* < 0.0001]. Dunn’s *post-hoc* multiple comparison further revealed that distance traveled in WAZM open arms by *affected* group was less than both *control* (*p* < 0.0001) and *unaffected* animals (*p* < 0.0001) (Figure 3F). No main effect of group was found by ordinary one-way ANOVA in the (v) *total time spent freezing in WAZM* across the 3 groups (Figure 3G). In the EPM test, significant main effect of group was found in (vi) *total time spent freezing in EPM* using ordinary one-way-ANOVA test across the three groups [F_(2,106)_ = 35.13, *p* < 0.0001] Bonferroni’s multiple comparison test showed increased freezing of *affected* animals than *control* (*p* < 0.0001) and *unaffected* (*p* < 0.0001) animals (Figure 3H). Kruskal–Wallis test showed significant difference between groups in (vii) *anxiety index in closed arms* of EPM [H (3) = 19.06, *p* < 0.0001]. Dunn’s *post-hoc* test revealed *affected* animals had an increased anxiety index as compared to *control* (*p* < 0.05) and *unaffected* animals (*p* < 0.001) (Figure 3I). In all the parameters no significant difference was found in the behavior between the *control* and *unaffected* animals. This showed that the un*affected* animals were behaviorally similar to control animals. The observation that the *trauma-affected* animals had worse average group effects on behavioral parameters than *controls* and *unaffected animals* reinforced the fact that IBP1 effectively categorized *affected* and *unaffected* animals post-UWT.

#### Individual behavioral profiling-1 efficiently identified treatment-responders from treatment non-responders after fluoxetine treatment

**FIGURE 4:**
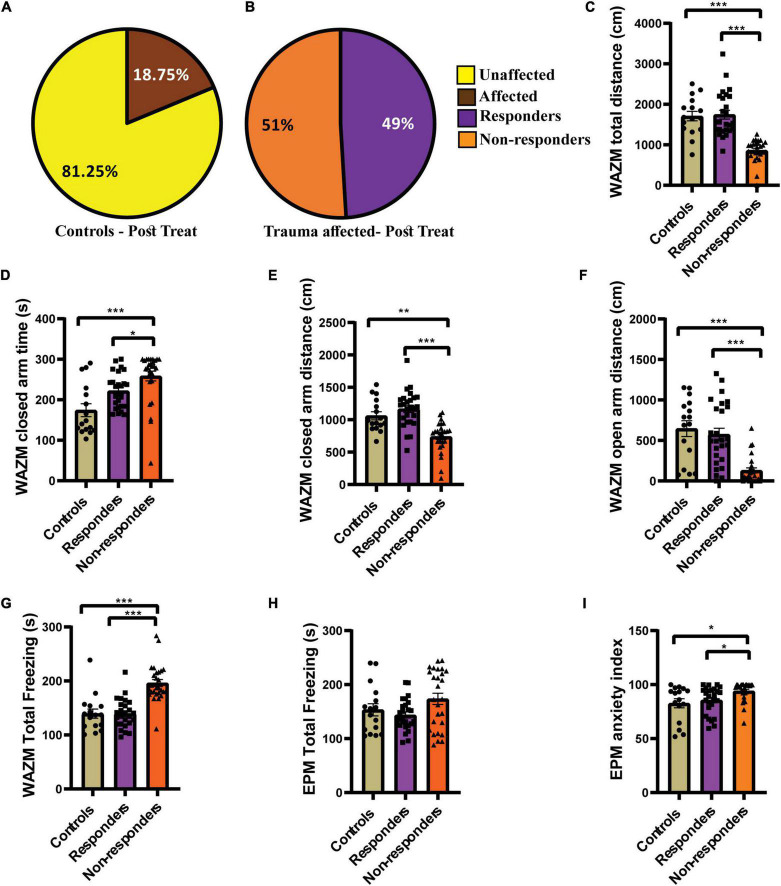
IBP1 classified JVS+UWT *affected* animals as treatment *responders* and *non-responders*. **(A)** Percentage of *affected* and *unaffected* animals in the *control* group at the post-fluoxetine time point. **(B)** Percentage distribution of treatment *responders* and *non-responders* in the trauma *affected* group after fluoxetine treatment. Controls (*n* = 16), trauma *affected* animals administered fluoxetine (*n* = 53). **(C)** Total distance traveled in WAZM by the *non-responders* was the least while *responders* and *control* traveled similar distance [Kruskal–Wallis test (H (3) = 41.51, *p* < 0.0001); Dunn’s *post-hoc* comparison p*** < 0.0001]. **(D)** Time spent in the WAZM closed arms was the highest by the *non-responders* but *responders* and *controls* spent similar time [Kruskal–Wallis test (H (3) = 20.62, *p* < 0.0001); Dunn’s *post-hoc* comparison p*** < 0.0001, p* < 0.05)]. **(E)** Distance traveled in the closed arms was the least by *non-responders* but *responders* and *controls* traversed similar distance [Kruskal–Wallis test (H (3) = 28.11, *p* < 0.0001); Dunn’s *post-hoc* comparison p** < 0.01, p*** < 0.0001]. **(F)** Distance traveled in the open arm of the WAZM was least by the *non-responders* while *responders* and *controls* covered similar distances [Kruskal–Wallis test (H (3) = 27.21, *p* < 0.0001); Dunn’s *post-hoc* comparison p*** < 0.00001]. **(G)** Total time spent freezing in the WAZM was highest amongst the *non-responders* but *responders* and *controls* spent similar time freezing [Kruskal–Wallis test (H (3) = 34.13, *p* < 0.0001); Dunn’s *post-hoc* comparison p*** < 0.00001]. **(H)** Time spent freezing in the EPM was not statistically different between *controls, responders* and *non-responders* [Kruskal–Wallis test (H (3) = 3.573, *p* = ns]. **(I)** Anxiety index in the EPM was highest in the *non-responders* while *responders* and *controls* had similar anxiety index [Kruskal–Wallis test (H (3) = 11.85, *p* < 0.01); Dunn’s *post-hoc* comparison p* < 0.05]. All data represented as mean ± SEM. *Controls* (*n* = 16), treatment *responders* (*n* = 26), treatment *non-responders* (*n* = 27).

Individual behavioral profiling-1 successfully identified animals that responded to the fluoxetine treatment. Around 49% of the trauma *affected* animals that received fluoxetine treatment showed recovery in ≥4 out of 7 behavioral parameters (Figure 4B). Fifty-one of the trauma-affected animals did not show recovery and were classified as *non-responder* (Figure 4B). This finding corroborated human statistical data indicating that only a subset of PTSD patients respond to therapy. IBP-1 also profiled the control animals as *affected* or *unaffected* with the percentage of *unaffected* animals much higher than *affected* animals (Figure 4A).

The behavior profiling was further supported by the differences observed in the group average effect of *responders* and *non-responders* on behavior. The average group effect of the treatment *responders* on most behavioral parameters was either found to be similar to healthy *control* animals or better than the *non-responders*. Kruskal–Wallis test showed significant difference between the three groups in the (i) *total distance traveled in WAZM* [H (3) = 41.51, *p* < 0.0001]. Dunn’s *post-hoc* comparison revealed that the non-*responders* traveled significantly less distance than both *controls* (*p* < 0.0001) and *responders* (*p* < 0.00001). The *responders* and *controls* traversed similar distance in the WAZM (Figure 4C). Similarly, significant main effect of groups was found in (ii) *time spent in closed arms* as compared by Kruskal–Wallis test [H (3) = 20.62, *p* < 0.0001]. Dunn’s *post-hoc* comparison revealed that *non-responders* spent significantly more time in the closed arms of the WAZM than *controls* (*p* < 0.00001) and *responders* (*p* = 0.0151). No significant difference was found in the time spent in closed arms between the *controls* and *responders* (Figure 4D). The average group effect was measured for (iii) *distance traveled in closed arms of WAZM* using Kruskal–Wallis test and was found to be significantly different between the groups [H(3) = 28.11, *p* < 0.00001]. Dunn’s *post-hoc* comparison showed that *non-responders* traversed significantly less distance in the closed arms of the WAZM than *controls* (*p* = 0.0019) and *responders* (*p* < 0.0001). *The controls* and *responders* traversed similar distance in the closed arms and no significant difference was found between them (Figure 4E). Significant group differences were also found in the iv) *distance traveled in the open arm of the WAZM* as analyzed by Kruskal–Wallis test [H (3) = 27.21, *p* < 0.0001]. Dunn’s *post-hoc* comparison revealed that the *non-responders* traveled significantly less distance in the open arm as compared to *controls* (*p* < 0.00001) and *responders* (*p* < 0.00001). No significant difference was found between the *controls* and *responders* in the distance traveled in the open arms (Figure 4F). Similar trend was observed in the (v) *total time spent freezing* in the WAZM by the three groups. Significant main effect of groups was found in Kruskal–Wallis test [H (3) = 34.13, *p* < 0.0001) and Dunn’s *post-hoc* comparison showed that the *controls* are behaviorally same as *responders* but the *non-responders* spent significantly more time freezing than the *controls* (*p* < 0.00001) and *responders* (*p* < 0.00001) (Figure 4G). No significant main effect of groups was found in the (vi) *total time spent freezing* in the EPM between the groups (Figure 4H). However, significant differences was found between the groups in the (vii) *anxiety index time in EPM* as measured by Kruskal–Wallis test [H (3) = 11.85, *p* < 0.01]. Dunn’s *post-hoc* comparison further revealed that the non-*responders* had significantly higher anxiety index than the *controls* (*p* = 0.0117) and *responders* (*p* = 0.0102). No significant difference was found in the anxiety index between the *controls* and *responders* (Figure 4I). The results clearly showed that behaviorally, *treatment-responders* were similar to *controls*, while *non-responders* continued to perform worse in all behavioral parameters than both *controls* and *responders*.

#### Biochemical proof of concept of individual behavioral profiling-1: Changes in GABA_A_α2, GluN1, and GluN2A expression in the ventral hippocampus after treatment

##### vDG

**FIGURE 5:**
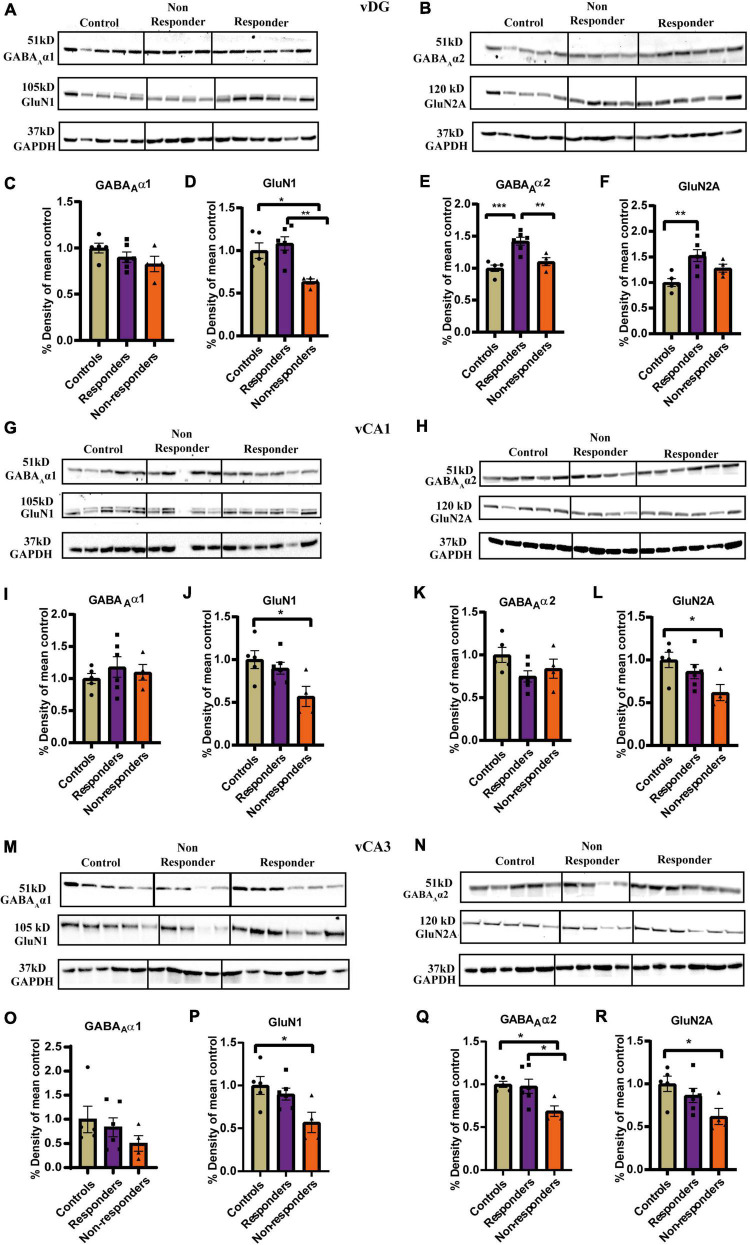
Experssion profile of GABA_A_α1, GABA_A_α2, GluN1, and GluN2A in the ventral hippocampus between *controls*, treatment *responders* and *non-responders*. **(A)** Gel images of GABA_A_α1, GluN1, and GAPDH for *controls*, treatment *responders* and *non-responders* in the vDG. **(B)** Gel images of GABA_A_α2, GluN2A, and GAPDH for *controls*, treatment *responders* and *non-responders* in the vDG. **(C)** GABA_A_α1 expression level was similar between the theree groups in vDG [Ordinary one-way ANOVA (F_(2,12)_ = 1.717, *p* = ns)]. **(D)** GluN1 levels was the least in the *non-responders* while *controls* and *responders* had almost similar level of expression in vDG [Ordinary one-way ANOVA (F_(2,12)_ = 8.486, *p* = 0.005); Bonferroni’s *post-hoc* test p* < 0.05, p** < 0.01]. **(E)** GABA_A_α2 levels were highest in the *responders* while *controls* and *responders* showed similar level of expression in vDG [Ordinary one-way ANOVA (F_(2,12)_ = 15.15, *p* = 0.0005); Bonferroni’s *post-hoc* test p** < 0.01, p*** < 0.0001]. **(F)** GluN2A expression in the *responders* was significantly higher than *controls* but no significant difference was found with respect to *non-responders* in vDG [Ordinary one-way ANOVA (F_(2,12)_ = 7.679, *p* = 0.0071); Bonferroni’s *post-hoc* test p** < 0.01]. **(G)** Gel images of GABA_A_α1, GluN1 and GAPDH for *controls*, treatment respnders and *non-responders* in the vCA1. **(H)** Gel images of GABA_A_α2, GluN2A, and GAPDH for *controls*, treatment respnders and *non-responders* in the vCA1. **(I)** GABA_A_α1 expression level was similar between the theree groups in vCA1 [Ordinary one-way ANOVA (F_(2,12)_ = 0.4875, *p* = ns)]. **(J)** GluN1 expression levels of *non-responders* was significantly less than the *controls* in vCA1 [Ordinary one-way ANOVA (F_(2,12)_ = 5.054, *p* = 0.0256); Bonferroni’s *post-hoc* test p* < 0.05]. The levels of GluN1 between *responders* and *non-responders* though not statistically significant showed a strong trend (*p* = 0.093). **(K)** GABA_A_α2 expression level was similar between the theree groups in vCA1 [Ordinary one-way ANOVA (F_(2,12)_ = 2.410, *p* = ns)]. **(L)** GluN2A expression levels of *non-responders* was significantly less than the *controls* in vCA1 [Ordinary one-way ANOVA (F_(2,12)_ = 4.150, *p* = 0.0427); Bonferroni’s *post-hoc* test p* < 0.05]. The levels of GluN1 between *responders* and *controls* were similar and no statistically significant difference was found between *responders* and *non-responders*. **(M)** Gel images of GABA_A_α1, GluN1, and GAPDH for *controls*, treatment respnders and *non-responders* in the vCA3. **(N)** Gel images of GABA_A_α2, GluN2A and GAPDH for *controls*, treatment respnders and *non-responders* in the vCA3. **(O)** GABA_A_α1 expression level was similar between the theree groups in vCA3 [Ordinary one-way ANOVA (F_(2,12)_ = 1.015, *p* = ns)]. **(P)** GluN1 expression levels of *non-responders* was significantly less than the *controls* in vCA3 [Ordinary one-way ANOVA (F_(2,12)_ = 5.054, *p* = 0.0256); Bonferroni’s *post-hoc* test p* < 0.05]. The levels of GluN1 between *responders* and *non-responders* though not statistically significant showed a strong trend (*p* = 0.093). **(Q)** GABA_A_α2 levels was the least in the *non*-respnders while *controls* and *responders* had almost similar level of expression in vCA3 [Ordinary one-way ANOVA (F_(2,12)_ = 5.771, *p* = 0.0175); Bonferroni’s *post-hoc* test p* < 0.05]. **(R)** GluN2A expression levels of *non-responders* was significantly less than the *controls* in vCA3 [Ordinary one-way ANOVA (F_(2,12)_ = 4.150, *p* = 0.0427); Bonferroni’s *post-hoc* test p* < 0.05]. The levels of GluN1 between *responders* and *controls* were similar and no statistically significant difference was found between *responders* and *non-responders*. All data represented as mean ± SEM. *Controls* (*n* = 5), treatment *responders* (*n* = 6), treatment *non-responders* (*n* = 4).

No significant main effect of groups was found on the expression of GABA_A_α1 in vDG (Figures 5A,C,D). The receptor subunit expression of GABA_A_α2, GluN1 and Glun2A was found to be significantly altered between *controls*, *responders* and *non-responders* (Figures 5B,D–F). Expression patterns of GABA_A_α2 revealed significant differences between the groups [*F*_(2, 12)_ = 15.15, *p* = 0.0005]. Bonferroni’s *post-hoc* test showed that the *responders* had higher expression levels of α2 than both *controls* (*p* = 0.0006) and non-*responders* (*p* = 0.0087). No significant difference was found between *controls* and *non-responders* (Figure 5E). This demonstrated that, in contrast to *controls* and *non-responders*, the *responders* regulated the GABA_A_α2 expression differently that likely evoked a unique adaptive response in them. Ordinary-one way ANOVA revealed significant differences in the expression pattern of GluN1 between the groups [F_(2, 12)_ = 8.486, *p* = 0.005]. Bonnferoni’s *post-hoc* test revealed that the *responders* had similar expression pattern as control animals. The non-*responders* had lower expression of GluN1 than *controls* (*p* = 0.0264) and *responders* (*p* = 0.005) (Figure 5C). The result indicated that the *responders* regulated their GluN1 levels as *controls*, which the *non-responders* failed to do and hence were unable to elicit response to fluoxetine. Statistically significant difference in the expression patterns of GluN2A was found between groups by ordinary-one way-ANOVA [*F*_(2, 12)_ = 7.679, *p* = 0.0071] (Figures 5F–H). Bonferroni’s *post-hoc* test revealed that *responders* had significantly higher levels of GluN2A than *controls* (*p* = 0.0061) indicating a unique adaptive response. Though the average expression level of GluN2A of *non-responders* was lesser than that of *responders*, no significant statistical difference was found between the *non-responders* and *responders* (*p* = 0.3390) or *controls* (*p* = 0.2485).

##### vCA1

In vCA1 expression pattern of GABA_A_α1(Figure 5I) and GABA_A_α2 (Figure 5K) was not found to be significantly different between the three groups as analyzed by ordinary-one-way-ANOVA. However, expression levels GluN1 [*F*_(2,12)_ = 5.054, *p* = 0.0256] (Figure 5J) and GluN2A [*F*_(2,12)_ = 4.150, *p* = 0.0427] (Figure 5L) were found to be significantly different across groups when compared by ordinary-one way-ANOVA. Bonferroni’s *post-hoc* test revealed that the non-responders had significantly lower expression of GluN1 than controls (*p* = 0.0291) and also exhibited a similarly strong trend of lower expression when compared to responders (*p* = 0.093) (Figure 5J). The expression profile of GluN1 was similar between responders and controls (Figure 5J). Expression of GluN2A was significantly lower in non-responders than controls (*p* = 0.0428). There was no significant difference in the expression profile of responders compared to controls or non-responders (Figure 5L). Together the results suggested that the anxiety-like behavior in the non-responders may partially be due to low expression levels of GluN1 and GluN2A which they failed to normalize after fluoxetine treatment.

##### vCA3

GABA_A_α1 expression levels was not found to be significantly altered between the groups as revealed by Kruskal–Wallis test (Figures 5M–O). However, expression of GABA_A_α2 was significantly altered between groups [*F*_(2, 12)_ = 5.771, *p* = 0.0175]. Bonferroni’s *post-hoc* comparison showed that the non-*responders* had significantly lower expression levels than *controls* (*p* = 0.0293) and *responders* (*p* = 0.0355). Expression level between *controls* and *responders* was found to be similar (Figure 5Q). This indicated that the *responders* had a different expression pattern of α2 from the *non-responders* which highlights a biochemical difference between the two groups. Further, inability of *non-responders* to rescue the expression of GABA_A_α2 to the levels similar to *controls* and *responders* might had contributed to the failure to respond to fluoxetine. GluN1 expression levels was found to be significantly altered between the groups as measured by ordinary-one way-ANOVA [*F*_(2, 12)_ = 5.054, *p* = 0.0256]. Bonferroni *post-hoc* test revealed that the *non-responders* had significantly lower expression levels than *controls* (*p* = 0.0291). A similar trend was also observed between *non-responders* and *responders* (*p* = 0.0930) (Figure 5P). Expression levels of GluN2A was found to be significantly different between the groups by ordinary-one way-ANOVA [*F*_(2, 12)_ = 4.150, *p* = 0.0427]. Bonferroni’s *post-hoc* comparison showed *non-responders* had significantly lower GluN2A expression than *controls* (*p* = 0.0428). There was no significant difference in the expression profile of *responders* compared to *controls* or *non-responders* (Figure 5R). Decreased expression of GluN1 and GluN2A in the *non-responders* might be contributing factor to its inability to respond to treatment. The average expression of GluN1 and GluN2 responders though statistically insignificant was approximately higher by ∼35 % than the non-responders.

### Results experiment 2

#### Individual behavioral profiling-2 effectively distinguished trauma-affected from unaffected individuals, as verified by average behavioral performance of affected individuals

**FIGURE 6:**
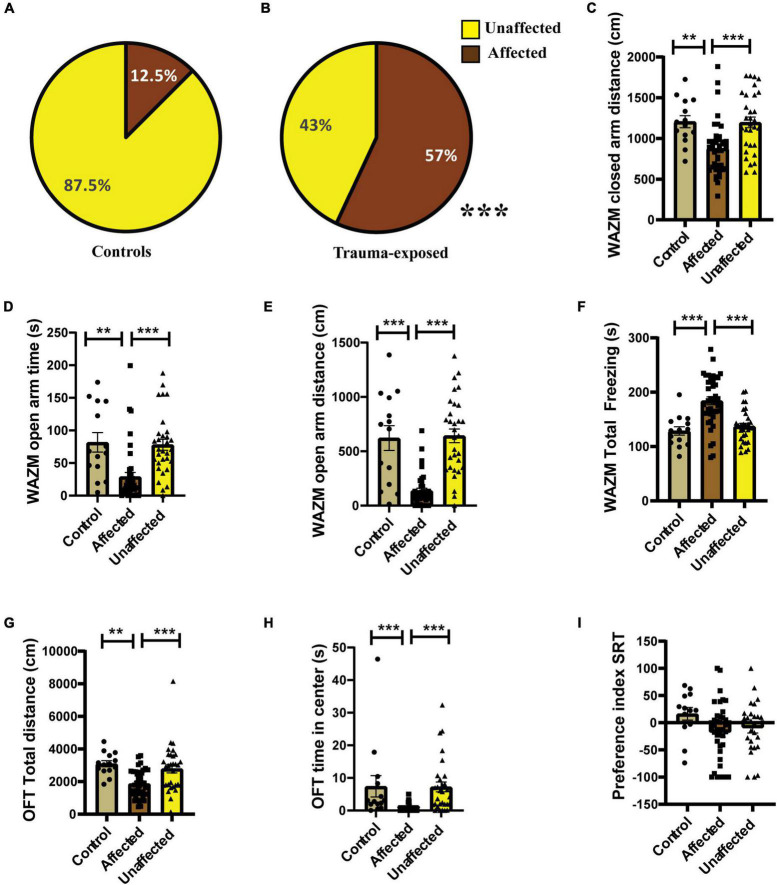
Individual behavioral profiling-2 (IBP2) classified animals into *affected* or *unaffected* in different groups. **(A)** Percentage distribution of *affected* animals and *unaffected* animals in *control* group. **(B)** Percentage of *affected animals* was found to be significantly higher in *trauma-exposed* animals than *controls* at 2 weeks post UWT [(Fisher exact test, p*** < 0.001); *Control* (*n* = 16), *trauma-exposed* (*n* = 72)]. **(C)** Distance traveled in the closed arms of WAZM was least in the trauma *affected* animals [Kruskal–Wallis test H (3) = 18.25, (*p* = 0.0001); Dunn’s *post-hoc* test p** < 0.01, p*** < 0.0001]. **(D)** Time spent in the open arms of WAZM was less by the trauma *affected* animals than *controls* and trauma un*affected* animals [Kruskal–Wallis test H (3) = 27.03, (*p* < 0.0001); Dunn’s *post-hoc* test p** < 0.01, p*** < 0.0001]. **(E)** Distance traveled in the open arms of WAZM was least in the trauma *affected* animals [Kruskal–Wallis test H (3) = 39.43, (*p* < 0.0001); Dunn’s *post-hoc* test p*** < 0.0001]. **(F)** Total time spent freezing in WAZM was highest by the trauma *affected* animals [Ordinary one-way ANOVA (F_(2,85)_ = 17.69, *p* < 0.0001); Bonferroni’s *post-hoc* test p*** < 0.0001]. **(G)** Total distance travelled in the OFT was least by the trauma *affected* animals [Kruskal–Wallis test H (3) = 21.22, (*p* < 0.0001); Dunn’s *post-hoc* test p** < 0.01, p*** < 0.001]. **(H)** Time spent in the anxiogenic canter of the OFT was least by the trauma *affected* animals [Kruskal–Wallis test H (3) = 33.98, (*p* < 0.0001); Dunn’s *post-hoc* test p*** < 0.001]. **(I)** Preference index for unfamiliar animal in the SRT was not significantly different between the three groups. The *controls* and trauma *unaffected* individuals had no statistically different performance in any of the behavioral parameters [Ordinary-one way-ANOVA (*F*_(2, 85)_ = 2.210, *p* = ns)]. All values are mean ± SEM. Controls (*n* = 16), trauma *affected* (*n* = 41), trauma *unaffected* (*n* = 31).

Individual behavioral profiling-2 revealed that 57% of trauma-exposed animals were *affected* (Figure 6B), compared to 12.5% of *controls* (Figure 6A). According to Fisher exact test, the percentage of *affected* animals in the JVS+UWT group was significantly greater than the *controls* (*p* = 0.0017). The distribution of *affected* and *unaffected* individuals obtained using *IBP-2* was compared to that obtained from IBP-2 using Fisher exact test but no statistically significant difference was identified (*p* = 0.1423).

The average group effect on the 7 behavioral parameters was compared between the trauma *affected, unaffected* and *control* animals. Statistically significant difference was found in the (i) *distance traveled in the closed arms of WAZM* between the groups [H(3) = 18.25, *p* = 0.0001], as measured by Kruskal–Wallis test. Dunn’s *post-hoc* multiple comparison showed that the *affected* individual traveled significantly less distance in closed arms than *controls* (*p* = 0.0039) and *unaffected* animals (*p* = 0.0006). The *controls* and *unaffected* animals traveled similar difference in the closed arms of WAZM and no significant difference was found between the two groups (*p* > 0.0999) (Figure 6C). (ii) *Time spent in the open arms of the WAZM* was compared using Kruskal–Wallis test and was found to be significantly different across groups [H(3) = 27.03, *p* < 0.0001]. Dunn’s *post-hoc* multiple comparison showed that the *affected* animals spent significantly less time in the anxiogenic open arms than the *controls* (*p* = 0.0011) and *unaffected* animals (*p* < 0.00001). The *controls* and *unaffected* animals spent similar time in the open arms of the WAZM with no significant statistical difference found between them (*p* > 0.0999) (Figure 6D). (iii) *Distance traveled in the open arms of the WAZM* was found to be significantly different between groups when measured using Kruskal–Wallis test [H(3) = 39.43, *p* < 0.0001). Dunn’s *post-hoc* test showed that the *affected* animals traveled less distance in the anxiogenic open arms than *controls* (*p* = 0.0001) and *unaffected* animals (*p* < 0.00001). No significant difference was found between the *controls* and *unaffected* animals (*p* > 0.9999) and both the groups appeared to travel similar distance in the open arms of WAZM (Figure 6E). Statistically significant difference was found between groups in the (iv) *total time spent freezing in the WAZM* using ordinary-one way-ANOVA and was found to be significantly different between groups [*F*_(2,82)_ = 17.69, *p* < 0.0001]. Bonferroni’s *post-hoc* test revealed that the *affected* individual spent significantly more time freezing in the WAZM than *controls* (*p* < 0.00001) and *unaffected* animals (*p* < 0.00001). No significant difference was found between the *controls* and *unaffected* individual in terms of total time spent freezing in the WAZM (*p* > 0.9999) (Figure 6F). (v) *Total distance covered in the OFT* was compared between the three groups using Kruskal–Wallis test and was found to be significantly different [H(3) = 21.22, *p* < 0.00001]. Dunn’s *post-hoc* comparison test showed that the *affected* individuals traveled significantly less distance in the OFT as compared to *controls* (*p* = 0.0002) and *unaffected* animals (*p* = 0.0018). There was no significant difference in the *total distance traveled in OFT* between the *controls* and the *unaffected* (*p* = 0.5335) (Figure 6G). (vi) *Time spent in the center of OFT* was compared between groups and was found to be significantly different using Kruskal–Wallis test [H(3) = 33.98, *p* < 0.00001]. Dunn’s *post-hoc* comparison revealed that the *affected* individuals spent significantly less time in the anxiogenic center of the OFT than *controls* [*p* = 0.0003] and *unaffected* individuals [*p* < 0.00001]. No statically significant difference was found between the *controls* and *unaffected* (*p* > 0.9999). No significant difference was found between the groups in the (vii) *preference index to novel* animal in SRT using ordinary-one way-ANOVA [*F*_(2, 82)_ = 2.210, *p* = 0.1160] (Figures 6H,I).

Together, the results indicated that the behaviorally profiled *affected* animals displayed increased anxiety-like behavior. The animals which were profiled as *unaffected* by IBP-2 were behaviorally similar to the healthy *controls* giving further validation to the behavioral profiling approach.

#### Individual behavioral profiling-2 correctly identified treatment-responders from non-responders as verified by average behavioral performance of the responders

**FIGURE 7:**
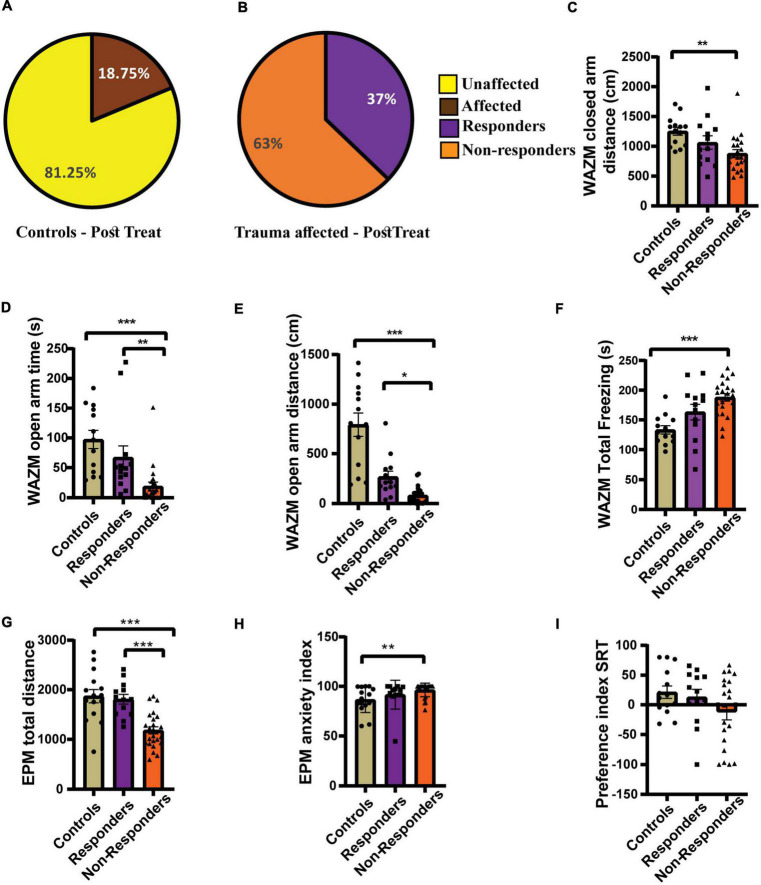
Individual behavioral profiling 2 (IBP2) classified JVS+UWT *affected* animals as treatment *responders* and *non-responders*. **(A)** Percentage of *affected* and *unaffected* animals in the *control* group at the post-fluoxetine time point. **(B)** Percentage distribution of treatment *responders* and *non-responders* in the JVS+UWT *affected* group after fluoxetine treatment. **(C)** Distance travelled in the closed arms of the WAZM was less by the *non-responders* than the *controls*. [Kruskal–Wallis test H (3) = 12.42, (*p* = 0.002); Dunn’s *post-hoc* test p** < 0.01]. No statistical significance was found between *responders* and *controls* or *non-responders.* **(D)** Time spent in the open arms of WAZM was less by the *non-responders* than both *controls* and *responders* [Kruskal–Wallis test H (3) = 22.99, (*p* < 0.0001); Dunn’s *post-hoc* test p** < 0.01, p*** < 0.0001]. *Responders* had no significant difference with *controls.* **(E)** Distance travelled in the open arm of the WAZM was least by the *non-responders* while *responders* and *controls* covered similar distances [Kruskal–Wallis test H (3) = 29.21, (*p* < 0.0001); Dunn’s *post-hoc* test p* < 0.05, p*** < 0.0001]. **(F)** Total time spent freezing in the WAZM was higher in the *non-responders* than *controls*. [Ordinary one-way ANOVA (*F*_(2,48)_ = 10.52, *p* = 0.0002); Bonferroni’s *post-hoc* test p*** < 0.0001]. No significant difference was observed in the time spent freezing in WAZM between *responders* and *controls* or *non-responders*. **(G)** Total distance travelled in the EPM was least in the *non-responders* while *responders* and *controls* spent similar distance [Ordinary one-way ANOVA (*F*_(2,48)_ = 17.23, *p* < 0.00001); Bonferroni’s *post-hoc* test p*** < 0.0001]. **(H)** Anxiety index in the EPM in the *non-responders* was higher than the *controls* [Kruskal–Wallis test H (3) = 10.85, (*p* = 0.0044); Dunn’s *post-hoc* test p** < 0.01]. No significant difference was observed between *responders* and *controls* or *non-responders*. **(I)** Preference index for unfamiliar animal in the SRT was not significantly different between the three groups *controls* [Kruskal–Wallis test (H (3) = 1.979, *p* = ns)]. All data represented as mean ± SEM. Controls (*n* = 16) *treatment* responders (*n* = 13), treatment *non-responders* (*n* = 22).

Individual behavioral profiling-2 successfully distinguished treatment *responders* from *non-responders* after fluoxetine therapy. Thirty-seven percent of the affected population was found to be treatment *responders* and 63% did not respond to the treatment (Figure 7B). The control population was also screened for any affected individuals, however the percentage of affectedness remained low at 18.5% (Figure 7A). No statistically significant difference was found in the distribution of *responders* and *non-responders* between experiment 1 and experiment 2 as compared by Fisher’s test (*p* = 0.2842).

The average group effect of the *responders, non-responders* and *controls* was measured for the 7 behavioral parameters chosen. (i) *Distance travelled in the closed arms of WAZM* was compared across groups using Kruskal–Wallis test and was found to be significantly different [H (3) = 12.42, *p* = 0.0020]. Dunn’s *post-hoc* comparison showed that the *non-responders* traveled significantly less than *controls* in the closed arms (*p* = 0.0013). The mean rank difference between *responders* and *controls* was 9.670, and between *responders* and *non-responders* was 7.524, although neither difference was statistically significant (*p* = 0.23367, *p* = 0.3967 respectively) (Figure 7C). (ii) *Time spent in the open arms* of the WAZM was found to be significantly different across groups using Kruskal–Wallis test [H (3) = 22.99, *p* < 0.00001). Dunn’s *post-hoc* test revealed no significant difference in the time spent in the anxiogenic open arms between the *responders* and *controls* (*p* = 0.8052). However, the *non-responders* spent significantly less time in the anxiogenic open arms of the WAZM than *controls* (*p* < 0.00001) and *responders* (*p* = 0.0036) (Figure 7D). iii) *Distance travelled in the open arms of the WAZM* was found to be significantly different between the groups as seen by Kruskal–Wallis test [H (3) = 29.21, *p* < 0.00001]. Further *post-hoc* comparison using Dunn’s test revealed that the non-*responders* travelled significantly less in the open arms than the *controls* (*p* < 0.00001) and the *responders* (*p* = 0.0141). No statistically significant difference was found between *responders* and *controls* (*p* = 0.0788) (Figure 7E). iv) *Total time spent freezing in the WAZM* was compared between the groups using ordinary-one way ANOVA and was found to be significantly different [*F*_(2, 48)_ = 10.52, *p* = 0.0002]. Bonferroni’s *post-hoc* test showed that the *non-responders* spent significantly more time freezing in the WAZM than the *controls* (*p* = 0.0001) (Figure 7F). (v) *Total distance traveled in EPM* was found to be significantly different between the groups as compared by ordinary-one way- ANOVA [*F*_(2, 48)_ = 17.23, *p* < 0.00001]. Bonferroni’s *post-hoc* test revealed that *non-responders* traveled significantly less distance in the EPM than *controls* (*p* < 0.00001) and *responders* (*p* = 0.0001). No statistically significant difference was observed between the *responders* and the *controls* (Figure 7G). (vi) *Anxiety index in the closed arms* of EPM was observed to be significantly different between the groups as measured by sung Kruskal–Wallis test [H(3) = 10.85, *p* = 0.0044]. Dunn’s *post-hoc* comparison showed that the *non-responders* exhibited significantly higher anxiety index than the *controls* (*p* = 0.0042). No significant difference was observed between *responders* and *controls* (*p* = 0.9197) or *non-responders* (*p* = 0.1622) (Figure 7H). No statistical difference was observed between the *responders* and *controls* (*p* = 0.0937) or *non-responders* (*p* = 0.1390). Kruskal–Wallis test revealed no significant difference between groups in the *vii) preference index for novel animal in SRT* (Figure 7I). The findings revealed that *responders* recovered in most behavioral parameters and were either behaviorally equivalent to *controls* or better than non-*responders*. This demonstrated that IBP-2 was effective in detecting *responders* within the fluoxetine-treated group.

#### Electrophysiological proof of concept of individual behavioral profiling-2: Increased local circuit activity in dDG of the responders

**FIGURE 8:**
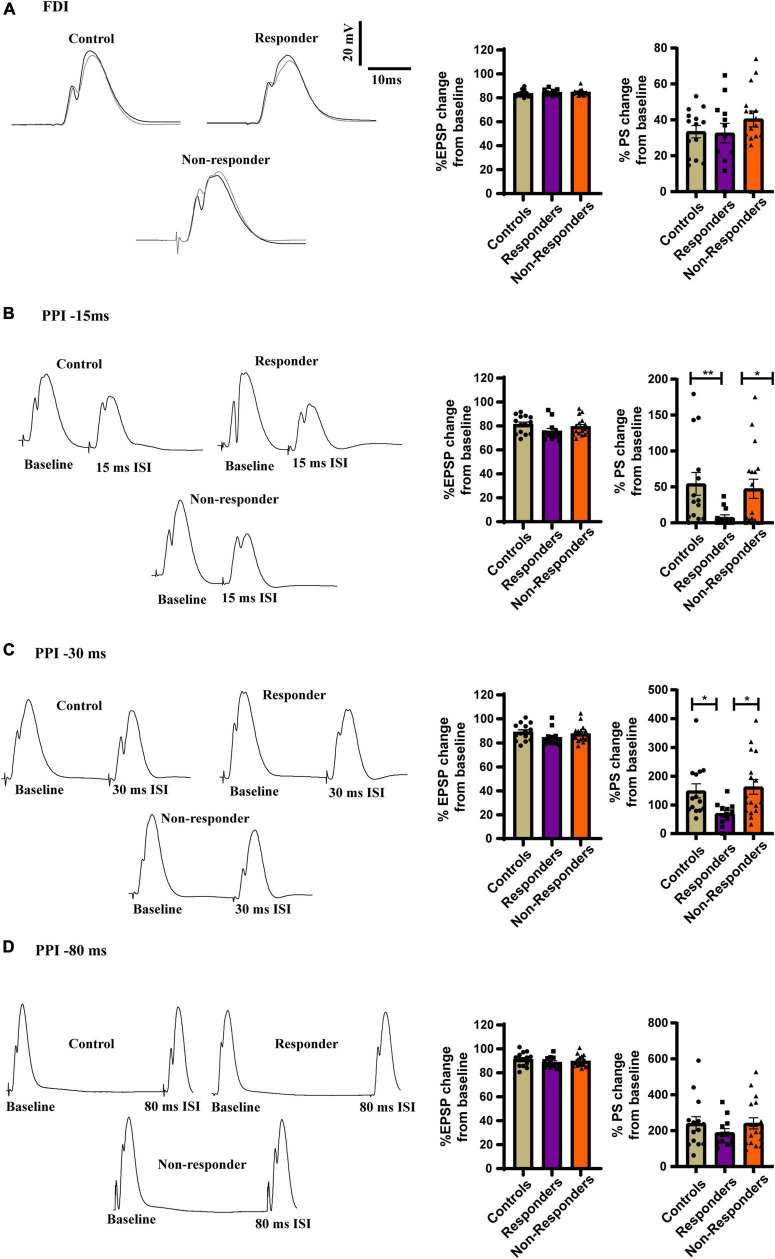
Increased inhibition in PPI at inter-stimulus interval of 15ms and 30 ms in *responders*. (**A**, left) Representative traces of FDI for *controls, responders* and *non-responders* showing feed-forward inhibition. (Right) Bar graphs of perecenatge change in EPSP slope [Kruskal–Wallis test (H(3) = 2.609, *p* = 0.2713)] and PS amplitude between the groups showed no change [Ordinary-one way-ANOVA (F_(2, 41)_ = 0.3740)]. (**B**, left) Representative traces of PPI at 15ms ISI for *controls, responders* and *non-responders* showing feed-back inhibition. (Right) Bar graph representing perecenatge change in EPSP slope did not show change any difference [Kruskal–Wallis test (H(3) = 4.230, *p* = 0.1207)]. Decreased PS amplitude in the *responders* than *controls* and *non-responders* indicated incrased feed-back inhibition the DG of the *responders* [Kruskal–Wallis test (H(3) = 10.27, *p* = 0.0059); Dunn’s *post-hoc* comparison p** < 0.01. p* < 0.05]. (**C**, left) Representative traces of PPI at 30 ms ISI for *controls, responders* and *non-responders*. Majority of *control* and *non-responders* showed fasciliation at 30 ms ISI while *responders* showed feed-back inhibition. (Right) Bar graph representing perecenatge change in EPSP slope did not show any difference between groups [Kruskal–Wallis test (H(3) = 3.792, *p* = ns)]. Decreased PS amplitude at 30ms ISI in the *responders* than *controls* and *non-responders* indicated incrased feed-back inhibition the DG of the *responders* [Kruskal–Wallis test (H(3) = 9.366, *p* = 0.0093); Dunn’s *post-hoc* comparison p* < 0.05]. (**D**, left) Representative traces of PPI at 80ms ISI for *controls, responders* and *non-responders* showing fasciltation. (Right) Bar graphs of perecenatge change in EPSP slope [Ordinary one-way ANOVA (*F*_(2,41)_ = 0.7357, *p* = 0.4857)] and PS amplitude between the groups showed no change [Ordinary one-way ANOVA (*F*_(2,41)_ = 0.7695, *p* = 0.4700)]. *Controls* (*n* = 14), treatment *responders* (*n* = 12) and treatment *non-responders* (*n* = 18).

The feed-forward inhibition in the dDG was measured using FDI protocol but the EPSP slope was not found to be significant between the group as measured by Kruskal–Wallis test [H(3) = 2.609, *p* = 0.2713] (Figure 8A). No statistically significant difference was found in the FDI population spike (PS) amplitude between groups as revealed by ordinary-one way-ANOVA [*F*_(2, 41)_ = 0.3740] (Figure 8A). However increased feed-back inhibition was observed in the *responders* when the local circuit activity was measured using PPI protocol. PS amplitude after 15ms ISI of PPI protocol was significantly different between groups as seen by using Kruskal–Wallis test [H (3) = 10.27, *p* = 0.0059]. Dunn’s multiple comparison revealed that the *responders* had increased local-circuit inhibition after 15ms protocol in PPI as evinced by significantly low PS amplitude as compared to *controls* (*p* = 0.0066) and *non-responders* (*p* = 0.0373). No statistically significant difference was observed between *controls* and *non-responders* (*p* > 0.9999) (Figure 8B). PS amplitude induced by PPI 30 ms protocol was significantly different between groups [H (3) = 9.366, *p* = 0.0093]. Dunn’s multiple comparison showed that the *responders* has significantly reduced population spike induction than *controls* (*p* = 0.0268) and *non-responders* (*p* = 0.0161). Majority of *control* and *non-responder* animals showed facilitation in the local-circuit after 30ms ISI between pulses unlike most of the *responders* that exhibited feed-back inhibition. No statistically significant difference was observed between *controls* and *non-responders* (*p* > 0.9999). The EPSP slope induced by 15ms (Figure 8B) and 30 ms (Figure 8C) protocol of PPI were found to be non-significant between groups as measured by Kruskal–Wallis test [H(3) = 4.230, *p* = 0.1207] and [H(3) = 3.792, *p* = 0.1502], respectively. No significant difference was observed between groups in EPSP slope or PS amplitude induced by PPI 80ms protocol as measured by ordinary-one way-ANOVA [*F*_(2,41)_ = 0.7357, *p* = 0.4857] and [F_(2,41)_ = 0.7695, *p* = 0.4700], respectively (Figure 8D).

No differences in EPSP or PS amplitude was discovered after TBS in LTP induction protocol as well (Supplementary Figure 2). Together, increased feed-back inhibition in the dDG local circuit was displayed only by the *responders* and was restricted to decreased PS amplitude in PPI 15 and PPI 30 protocol. This not only highlighted electrophysiological difference between the *responders* and the others but also highlighted a unique functional adaptive response of the *responders* to fluoxetine.

## Discussion

It is well-established that only a relatively small number of PTSD patients respond well to psychopharmacological treatment. It is estimated that only about 20-30% of them show full remission (Ravindran and Stein, 2010; Steckler and Risbrough, 2012). Despite that, the accepted approach of measuring efficacy of pharmacological treatments in animal models of psychiatric disorders refers to averaged group results (Katz and Hersh, 1981; Bodnoff et al., 1989; Rudolph and Feiger, 1999; Dulawa et al., 2004). Using group averages, and obscuring individual variability in treatment response in animal models significantly compromises their translational power. To address this drawback, we aimed in the current study to adapt a “Behavioral Profiling” analysis approach, originally developed for differentiating between trauma-exposed-*Affected* and trauma-exposed-*Unaffected* individuals (Ardi et al., 2016), in order to enable differentiating between treatment-*responders* and treatment-*non-responders* individuals.

We demonstrate here, for the first time, the effectiveness of employing this approach for assessing treatment efficacy in an animal model of PTSD. IBP 1 and IBP2 properly estimated that less than 50% of the treated animals were responsive to fluoxetine. These outcomes are consistent with PTSD patients’ overall response rate to SSRIs, which is around 60%, with only 20–30% of patients attaining full remission (Berger et al., 2009).

The results also serve to further support the efficacy of the ‘Behavioral Profiling’ approach in differentiating between *affected* and *Unaffected* individuals following exposure to trauma. Post-categorisation analysis clearly revealed that the group average effects of the trauma-*affected* animals were worse than those of controls and trauma-*unaffected* animals in the majority of IBP1 and IBP-2 behavioral characteristics, which included both exploratory and anxiety-like behaviors.

In a similar way, the majority of IBP1’s behavioral parameters also showed a clear distinction between *responders* and *non-responders*. The *non-responders* fared worse than both controls and *responders*, while the *responders* appeared to behave similarly to the controls. The *non-responders* were also found to be negatively affected in all behavioral parameters of IBP2 and performed worse than controls in all parameters with the exception of preference index in SRT. A distinct difference in performance was seen between the *responders* and *non-responders* in the time spent in open arms in the open arms of WAZM, total distance covered in the open arms of the WAZM and the total distance traveled in the EPM. In the other parameters the average group effect of the *responders*, though not significantly different, was closer to controls than the *non-responders*. These results supported the classification performed also by the IBP2. Additionally, we did not observe any difference in the percentage of affected population after trauma and percentage of fluoxetine responders, as assessed by IBP-1 or IBP-2. This is an important finding since it demonstrates the power of the ‘Behavioral Profiling’ approach. The classification of animals as trauma *affected* or *unaffected*, or treatment *responders* or *non-responders* is not dependent on the exact behavioral criteria used but rather on the principle approach. This gives our individual behavioral profile method an extremely adaptable advantage, enabling it to be expanded into other behavioral paradigms to assess the effectiveness of various therapies using a variety of animal models and behavioral tests.

The functional importance of the individual behavioral profiling technique was further proven by showing that the behavioral classification of animals into *responders* and *non-responders* is also associated with pertinent alterations in the E/I balance.

Biochemical investigation of E/I balance found a distinct difference between *responders* and *non-responders* by evaluating expression levels of receptor subunits of GABA_A_ and NMDAR. The most prevalent and significant difference between the *responders* and *non-responders* was in GABA_A_α2 expression levels in the vDG and vCA3. GABA_A_α2 has been linked to anxiolytic behavior (Pham et al., 2009; Raud et al., 2009; Wisłowska-Stanek et al., 2013) as well as facilitating anxiolytic effects of benzodiazepines and barbiturates (Low et al., 2000; Morris et al., 2006; Dixon et al., 2008). Polymorphism of GABA_A_α2 is also associated with childhood trauma and risk of developing PTSD in adulthood (Nelson et al., 2009). Additionally, earlier research from our group revealed elevated levels of GABA_A_α2 in the vCA1 of trauma-exposed but resilient animals (Ardi et al., 2016). Together, these studies indicate a neuroprotective role of GABA_A_α2. Increased levels of GABA_A_α2 in the vDG and vCA3 of the hippocampus in *responders* compared to *non-responders* may reflect a unique mechanism of functional adaptation in the *responders* in response to treatment that *non-responders* do not attain. The *responders* exhibited elevated levels of GABA_A_α2 in the vDG also compared with controls, showing that despite being behaviourally similar, the *responders* demonstrate active coping mechanisms, not found in controls, presumably enabling them to behaviourally respond well. No change in expression patterns of GABA_A_α1 was seen between the groups. GABA_A_α1 is the most abundant subunit of GABA_A_ receptor ∼60% (Benke et al., 2004) and may have intact global expression. However, further investigation is required to determine whether synapse or cell-specific changes have occurred.

The expression patterns of the NMDAR subunit changed concurrently amongst the groups, as well. In comparison to *responders, non-responders* had lower levels of GluN1 expression in the vDG. This difference between *responders* and *non-responders* further serves to functionally validate the significance of the behavioral profiling approach. Additionally, we observed that the ventral hippocampus of the *non-responders* consistently had lower levels of GluN1 expression than *controls*. Similar downtrend of expression was seen in the vCA1 and vCA3 as compared to the *responders*. Downregulation of GluN1 in the ventral hippocampus has been observed after chronic stress (Pacheco et al., 2017) and in hippocampus after prenatal stress (Sun et al., 2013). Therefore, lower GluN1 expression in *non-responders* might indicate a maladaptive change; nevertheless, further in-depth research is required in order to establish this possibility. In addition, Glun2A expression was lower in the vCA1 and vCA3 of *non-responders* compared to controls. Alteration in the levels GluN2A has been reported after stress and was related to anxiety like behaviors (Calabrese et al., 2012; Goulart et al., 2021). An increase in synaptic GuN1 and GluN2A in the hippocampus is also essential for dendritic arborisation, memory consolidation (Cercato et al., 2017) and contextual fear learning (Acutain et al., 2021). The most crucial fact is that GluN1 and GluN2A both have the potential to impact the induction of plasticity (Shipton and Paulsen, 2014; Baez et al., 2018; Zhou and Duan, 2018), and changes in their levels in *non-responders* are indicative of potential changes in synaptic plasticity which may lead to altered memory.

Together, the different pattern of alteration in the GABA_A_α2, GluN1, and GluN2A in the ventral hippocampus between *responders* and *non-responders* suggests a shift in E/I balance. Increased expression of GluN2A in the *responders*’ ventral DG compared to the controls further supports the idea that the *responders* are actively using coping mechanisms which involve E/I balance alterations, in order to establish a new stable state and return to normal behavioral patterns.

Electrophysiological investigation of the local circuit activity of the dorsal dentate gyrus further revealed indications for a shift in E/I balance in the *responders*. Antidepressant treatments, including fluoxetine, are known to alter local circuit activity in the dDG (Reisi et al., 2014). We did not observe any changes in feed-forward inhibition, as measured by FDI but did find altered PPI (Increased inhibition of the PS amplitude at 15 and 30 ms ISI), highlighting that changes in the local circuit in the *responders* may be specific to feedback inhibition. Increased inhibition of the PS amplitude of the granular cells is thought to be mediated through basket cells. It is considered that the inhibition induced during these shorter ISIs is mediated by fast acting GABA_A_ receptors, while longer ISIs result in the eventual recruitment of GABA_B_ receptors, leading to facilitation (Tuff et al., 1983; Albertson and Joy, 1987). We observed that at the 30ms ISI *controls* and *non-responders* mainly exhibited facilitation of the PS, but *responders* continued to show increased inhibition, again suggesting enhanced feedback inhibition in this group. Such alterations of the local circuit and of E/I balance, which were specific to *responders*, indicate changes in the computation properties of the region, resulting in altered processing of information in the dDG. Specific alterations in the GABA and NMDAR receptor subunits as well as specific changes in the local circuit activity solely in the *responders* point to the deployment of an active coping mechanism. We did not observe any changes in LTP between groups, suggesting that mechanisms of long-term memory storage may not have been affected, but further research is required in order to verify this.

Together, we show that behavioral categorisation into *responders* and *non-responders* is an important research approach, since it differentiates between different neural outcomes following drug treatment, as was exemplified by the differential effects on E/I balance both in the ventral and dorsal hippocampus. The results call for the need to adopt a “Behavioral Profiling” analysis approach when evaluating the efficacy and the neural outcome of drug treatment in animal models of psychiatric disorders.

## Data availability statement

The raw data supporting the conclusions of this article will be made available by the authors, without undue reservation.

## Ethics statement

The animal study was reviewed and approved by the Institutional Animal Care and Use Committee of the University of Haifa.

## Author contributions

IS: behavior experiments and analysis, western blots and analysis, analysis of electrophysiology experiments, and manuscript writing. MS-S: part of the electrophysiological experiments and analysis. AT and AB: part of the electrophysiological experiments. GR-L: supervising experiments and manuscript writing. All authors contributed to the article and approved the submitted version.
