# Supplementary material for: Individual behavioral profiling as a translational approach to assess treatment efficacy in an animal model of post-traumatic stress disorder
Source: Front Neurosci. 2022 Dec 22;16:1071482. doi: 10.3389/fnins.2022.1071482 (PMC9815535; doi:10.3389/fnins.2022.1071482)
Supplement: Supplementary file 1 [file Image_1.pdf]

Supplementary Material

1 Supplementary Figures

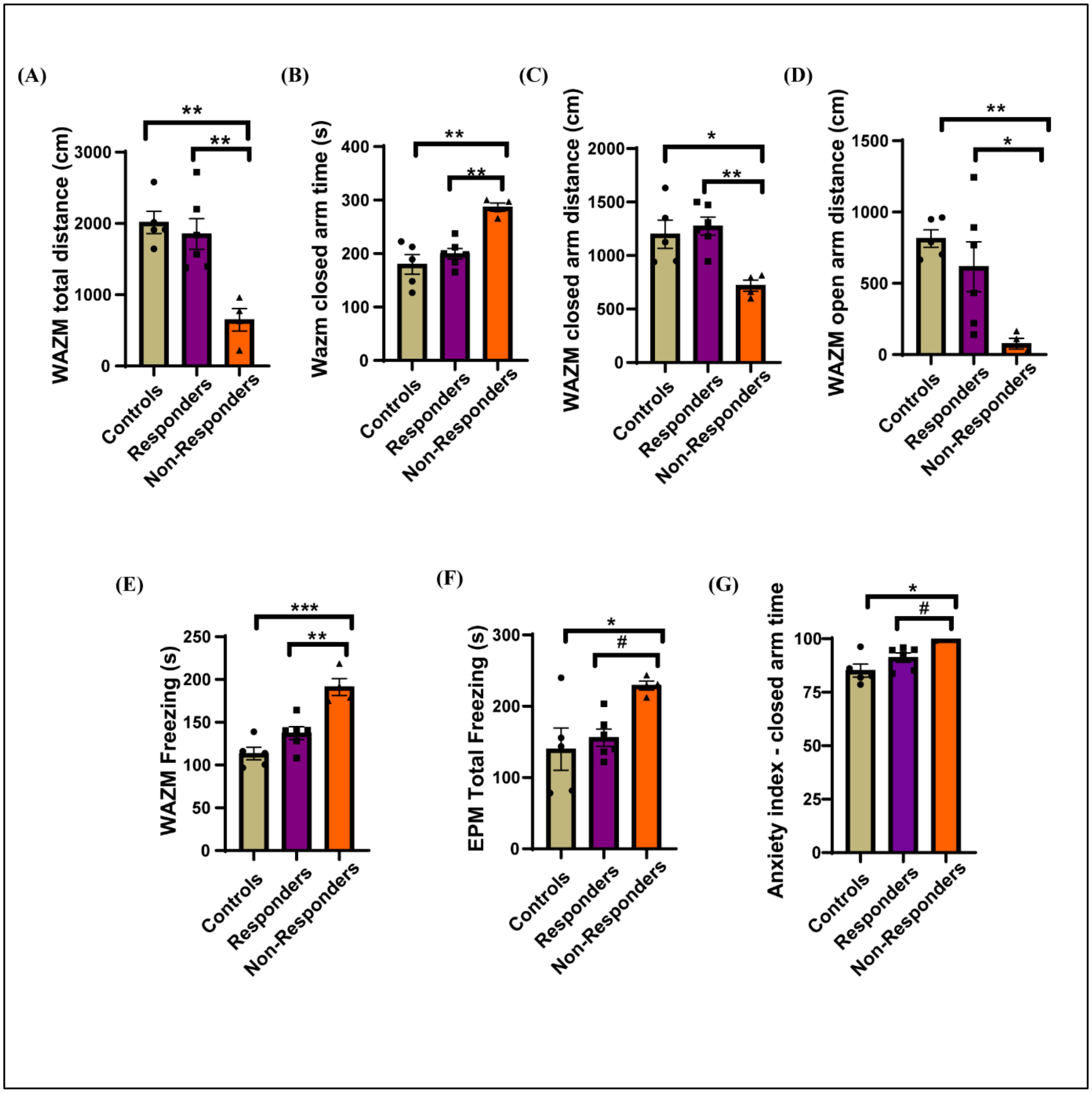

Supplementary Figure 1. Average group effect of *controls*, treatment *responders* and *non-responders* used in western blot experiments on behavioural parameters of WAZM and EPM. (A) Total distance travelled in WAZM by the *non-responders* was the least while *responders* and *control* travelled similar distance [Ordinary one-way ANOVA ($F_{(2,12)}$ = 13.51, p = 0.0008); Bonferroni's post hoc test p**<0.001]. (B) Time spent in the WAZM closed arms was the highest by the *non-responders* but

responders and *controls* spent similar time [Ordinary one-way ANOVA ($F_{(2,12)}$ = 16.05, p = 0.0004); Bonferroni's post hoc test p**<0.01 p***<0.0001]. (C) Distance travelled in the closed arms was the least by *non-responders* but *responders* and *controls* traversed similar distance [Ordinary one-way ANOVA ($F_{(2,12)}$ = 8.317, p = 0.0054); Bonferroni's post hoc test p*<0.05 p**<0.01]. (D) Distance travelled in the open arm of the WAZM was least by the *non-responders* while *responders* and *controls* covered similar istances [Ordinary one-way ANOVA ($F_{(2,12)}$ = 7.581, p = 0.0074); Bonferroni's post hoc test p*<0.05 p**<0.01]. (E) Total time spent freezing in the WAZM was highest amongst the *non-responders* but *responders* and *controls* spent similar time freezing [Ordinary one-way ANOVA ($F_{(2,12)}$ = 21.15, p = 0.0001); Bonferroni's post hoc test p**<0.01, p***<0.0001]. (H) Time spent freezing in the EPM was higher in the *non-responders* than the *controls* [Ordinary one-way ANOVA ($F_{(2,12)}$ = 5.293, p = 0.0225); Bonferroni's post hoc test p**<0.05, $p^{\#}$ = 0.0663]. A similarly strong trend was also observed between *responders* and *non-responders* (I) Anxiety index in the EPM was higher in the *non-responders* than the *controls*. A similarly strong trend was also observed between *responders* and *non-responders.* [Kruskal-Wallis test (H(3) = 9.165, p = 0.002); Dunn's post hoc test p**<0.01, $p^{\#}$ = 0.0843] All data represented as mean ± SEM. *Controls* (n = 5), treatment *responders* (n = 6), treatment *non-responders* (n = 4).

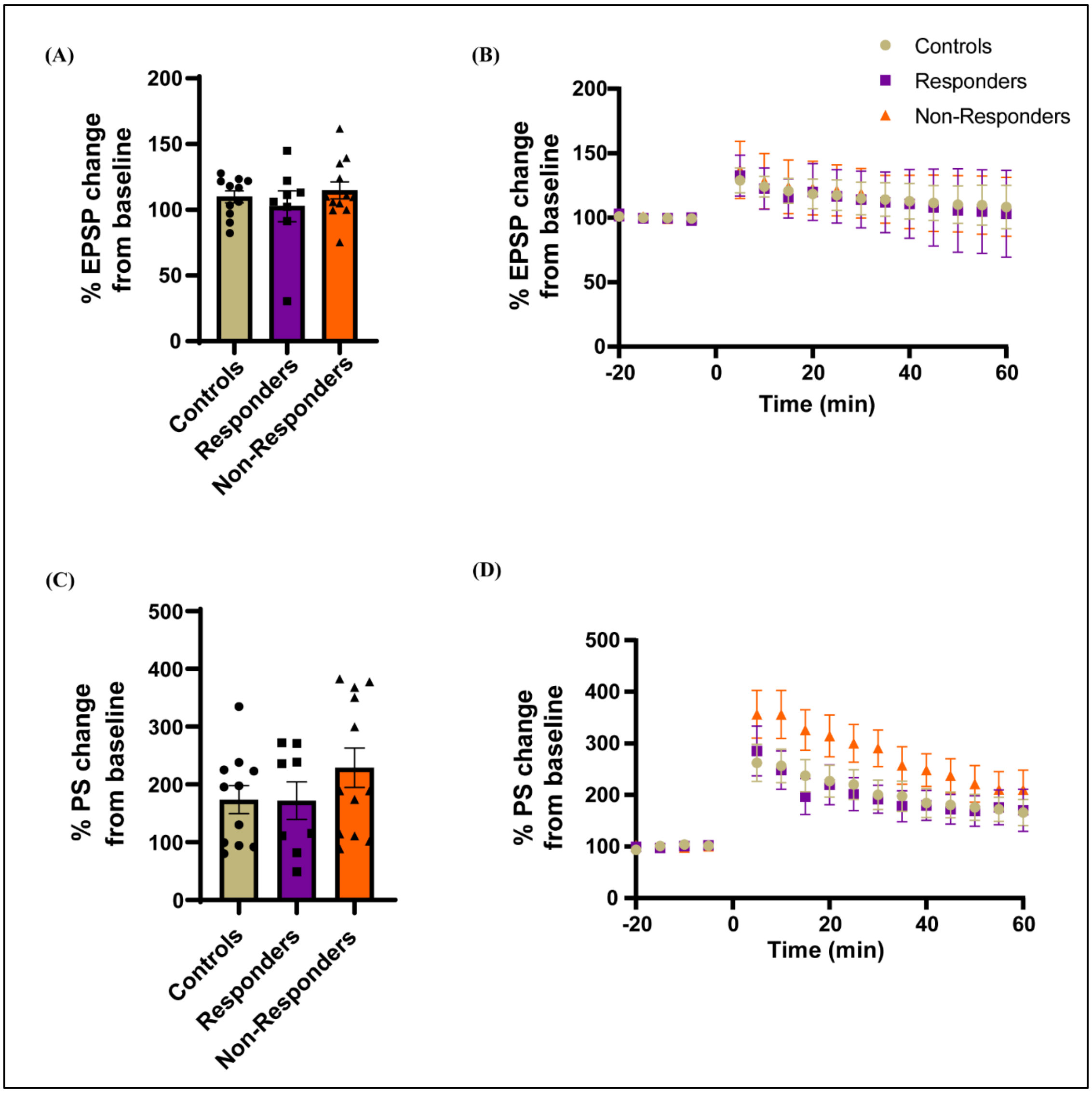

Supplementary Figure 2. (A) Sixty minutes following the application of TBS, *controls, responders* and *non-responders* showed increase in EPSP slope than baseline levels. (B) However no difference was observed between the groups across time in EPSP slope [2-way repeated measures, interaction effect ($F_{(22,319)}$= 0.8256, p = ns)] (C) The application of TBS also resulted in an increase in PS amplitude in *controls*, *responders* and *non-responders*. No difference was observed between the groups across time in PS amplitude [2-way repeated measures, interaction effect ($F_{22,319}$)= 1.443, p = ns)]. Controls (n = 11), responders (n = 8), non-responders (n = 13)
